# Supplementary material for: Heat adaptation of phage T7 under an extended genetic code
Source: Virus Evol. 2021 Dec 1;7(2):veab100. doi: 10.1093/ve/veab100 (PMC8923235; doi:10.1093/ve/veab100)
Supplement: veab100_Supp [file veab100_supp.zip › Sequencing_Analysis.zip!/Sequencing_Analysis/S1/evidence/JC_SIDE_1_47.html]

BRESEQ :: Evidence


| New junction evidence | | | | | | | | | | | |
| --- | --- | --- | --- | --- | --- | --- | --- | --- | --- | --- | --- |
|  | | seq id | position | reads (cov) | reads (cov) | score | skew | freq | annotation | gene | product |
| \* | ? | NC\_001604.1 | = 1256 | 0 (0.000) | 154 (0.040) | 67/574 | 22.6 | 99.7% | intergenic (–/–) | *–/–* | –/– |
|  | ? | NC\_001604.1 | 2737 = | 1 (0.000) |  |  |  |  | intergenic (–/–) | *–/–* | –/– |
| Rejected: Coverage evenness skew score above cutoff. | | | | | | | | | | | |

`TTTTCGACCACGCTTACGAAATGCTGAAAGAAAACATCCGTTATGATGACATCCGTGACACTGATGACCTGCACGATGCTATTCACATGGCTGCCGATAATGCAGTTCCGCACTACTACGCTGACATCTTTAGCGTAATGGCAAGTGAGGGCATTGACCTTGAGTTCGAAGACTCTGGTCTGATGCCTGACACCAAGGACGTAATCCGCATCCTGCAAGCGCGTATCTATGAGCAATTAACGATTGACCTCTGGGAAGACGCAGAAGACTTGCTCAATGAATACTTGGAGGAAGTCGAGGAGTACGAGGAGGATGAAGAGTAATGTCTAC  >  NC_001604.1/956‑1285`  

                                                                                                                                                                                                                                                                                                            `|`                               
`tttttgtccaaccTTACAAAATGCTGAAAGAAAAACTCCTTTATGATGAACTCCGTGACACTGATGACCTTCAAGATGCTATTCACATGGCTGCCGATAATGCAGTTCCGCACTACTACGCTGACATCTTTAGCGTAATGGCAAGTGAGGGCATTGACCTGGAGTTCGAAGACTCTGGTCTGATGCCTGACACCAAGGACGTAATCCGCATCCTGCAAGCGCGTATCTATGAGCAATTAACGATTGACCTCTGGGAAGACGCAGAAGACTTGCTCAATGAATACTTGGAGGAAGTCgagga                               <  1:620002/289‑1 (MQ=255)`  
`ttttCGACCACGCTTACGAAATGCTGAAAGAAAACATCCGTTATGATGACATCCGTGACACTGATGACCTGCACGATGCTATTCACATGGCTGCCGATAATGCAGTTCCGCACTACTACGCTGACATCTTTAGCGTAATGGCAAGTGAGGGCATTGACCTTGAGTTCGAAGACTCTGGTCTGATGCCTGACACCAAGGACGTAATCCGCATCCTGCAAGCGCGTATCTATGAGCAATTAACGATTGACCTCTGGGAAGACGCAGAAGACTTGCTCAATGAATACTTGGAGGAAGTCgagga                               <  1:390064/301‑1 (MQ=255)`  
`ttttCGACCACGCTTACGAAATGCTGAAAGAAAACATCCGTTATGATGACATCAGTGACACTGATGACCTGCACGATGCTATTCACATGGCTGCCGATAATGCAGTTCCGCACTACTACGCTGACATCTTTAGCGTAATGGCAAGTGAGGGCATTGACCTTGAGTTCGAAGACTCTGGTCTGATGCCTGACACCAAGGACGTAATCCGCATCCTGCAAGCGCGTATCTATGAGCAATTAACGATTGACCTCTGGGAAGACGCAGAAGACTTGCTCAATGAATACTTGGAGGAAGTCgagga                               <  1:507276/301‑1 (MQ=255)`  
`ttttCGACCACGCTTACGAAATGATGAAAGAAAACATCAGTTATGATGACATCCGTGAAACTGATGACCTGCACGATGCTATTCACATGGCTGCCGATAATGCAGTTCCGCACTACTACGCTGACATCTTTAGCGTAATGGCAAGTGAGGGCATTGACCTTGAGTTCGAAGACTCTGGTCTGATGCCTGACACCAAGGACGTAATCCGCATCCTGCAAGCGCGTATCTATGAGCAATTAACGATTGACCTCTGGGAAGACGCAGAAGACTTGCTCAATGAATACTTGGAGGAAGTCgagga                               <  1:668795/301‑1 (MQ=255)`  
`ttttCGACCACGCTGACGAAATGCTGAAAGAAAACATCCGTTATGATGACATCCGGGACACTGATGACCTGCACGATGCTATTCACATGGCTGCCGATAATGCAGTTCCGCACTACTACGCTGACATCTTTAGCGTAATGGCAAGTGAGGGCATTGACCTTGAGTTCGAAGACTCTGGTCTGATGCCTGACACCAAGGACGTAATCCGCATCCTGCAAGCGCGTATCTATGAGCAATTAACGATTGACCTCTGGGAAGACGCAGAAGACTTGCTCAATGAATACTTGGAGGAAGTCgagga                               <  1:523175/301‑1 (MQ=255)`  
 `tttCGACCACGCTTACGAAATTCTGAAATAAAACATCCGTTATGAGGACATCCGTGACACTGATGACCTGCACGTCTCTATTCACATGGTTGCCTATAATGCAGTTCCGCACTACTACGCTGACATCTTTAGCTTAATGGCAAGTGATGGCATTGACCTTGAGTTCGGAGACTCTGGTCTGATGCCTGACACCAAGGACGTAATCCGCATCCTGCAAGCGCGTATCTATGAGCAATTAACGATTGACCTCTGGGAAGACGCAGAAGACTTGCTCAATGAATACTTGGAGGAAGTCgaggaa                              <  1:591914/301‑2 (MQ=255)`  
 `tttCGACCACGCTTACGAAATGCTGAAAGAAAACATCCGTTATGATGACATCCGTGACACTGATGACCTGCACGATGCTATTCACATGGCTGCCGATAATGCAGTTCCGCACTACTACGCTGACATCTTTAGCGTAATGGCAAGTGAGGGCATTGACCTTGAGTTCGAAGACTCTGGTCTGATGCCTGACACCAAGGACGTAATCCGCATCCTGCAAGCGCGTATCTATGAGCAATTAACGATTGACCTCTGGGAAGACGCAGAAGACTTGCTCAATGAATACTTGGAGGAAGTCgaggaa                              <  1:218645/301‑2 (MQ=255)`  
 `tttCGACCACGCTTACGAAAGGCTGAAAGAAAACATCCGTTATGATGACATCCGTGACACTGATGACCTGCACGATGCTATTCACATGGCTGCCGATAATGCAGTTCCGCACTACTACGCTGACATCTTTAGCGTAATGGCAAGTGAGGGCATTGACCTTGAGGTCGAAGACTCTGGTCTGATGCCTGACACCAAGGACGTAATCCGCATCCTGCAAGCGCGTATCTATGAGCAATTAACGATTGACATCTGGGAAGACGCAGAAGACTTGCTCAATGAATACTTGGAGGAAGTCgaggaa                              <  1:295801/301‑2 (MQ=255)`  
 `tttCGACCACGCTAACGAAATGCTGAAAGAAAACATCCGTTATGATGACATCCGTGACACTGATGACCTGCACGATGCTATTCACATGGCTGCCGATAATGCAGTTCCGCACTACTACGCTGACATCGTTAGCGTAATGGCAAGTGAGGGCATTGACCTTGAGTTCGAAGACTCTGGTCTGATGCCTGACACCAAGGACGTAATCCGCATCCTGCAAGCGCGTATCTATGAGCAATTAACGATTGACCTCTGGGAAGACGCAGAAGACTTGCTCAATGAATACTTGGAGGAAGTCgaggaa                              <  1:189026/301‑2 (MQ=255)`  
 `ggagatgtgtataagagacaggCTGAAAGAAAACATCCGTTATGATGACATCCGTGACACTGATGACCTGCACGATGCTATTCACATGGCTGCCGATAATGCAGTTCCGCACTACTACGCTGACATCTTTAGCGTAATGGCAAGTGAGGGCATTGACCTTGAGTTCGAAGACTCTGGTCTGATGCCTGACACCAAGGACGTAATCCGCATCCTGCAAGCGCGTATCTATGAGCAATTAACGATTGACCTCTGGGAAGACGCAGAAGACTTGCTCAATGAATACTTGGAGGAAGTCgaggaa                              <  1:66905/280‑2 (MQ=255)`  
  `ttCGACCACGCTTACGAAATGCTGAAAGAAAACATCCGTTATGATGACATCCGTGACACTGATGACCTGCACGATGCTATTCACATGGCTGCCGATAATGCAGTTCCGCACTACTACGCTGACATCTTTAGCGTAATGGCAAGTGAGGGCATTGACCTTGAGTTCGAAGACTCTGGTCTGATGCCTGACACCAAGGACGTAATCCGCATCCTGCAAGCGCGTATCTATGAGCAATTAACGATTGACCTCTGGGAAGACGCAGAAGACTTGCTCAATGAATACTTGGAGGAAGTCgaggaag                             <  1:111138/301‑3 (MQ=255)`  
  `ttCGACCACGCTTACGAAATGCTGAAAGAAAACATCCGTTATGATGACATCCGTGACACTGATGACCTGCACGATGCTATTCACATGGCTGCCGATAATGCAGTTCCGCACTACTACGCTGACATCTTTAGCGTAATGGCAAGTGAGGGCATTGACCTTGAGTTCGAAGACTCTGGTCTGATGCCTGACACCAAGGACGTAATCCGCATCCTGCAAGCGCGTATCTATGAGCAATTAACGATTGACCTCTGGGAAGACGCAGAAGACTTGCTCAATGAATACTTGGAGGAAGTCgaggaag                             <  1:616102/301‑3 (MQ=255)`  
   `tCGACCACGCTTACGAAATGCTGAAAGAAAACATCCGTTATGATGACATCCGTGACACTGATGACCTGCACGATGCTATTCACATGGCTGCCGATAATGCAGTTCCGCACTACTACGCTGACATCTTTAGCGTAATGGCAAGTGAGGGCATTGACCTTGAGTTCGAAGACTCTGGTCTGATGCCTGACACCAAGGACGTAATCCGCATCCTGCAAGCGCGTATCTATGAGCAATTAACGATTGACCTCTGGGAAGACGCAGAAGACTTGCTCAATGAATACTTGGAGGAAGTCgaggaagt                            <  1:227398/301‑4 (MQ=255)`  
     `gACCACGCTTACGAAATGCTGAAAGAAAACATCCGTTATGATGACATCCGTGACACTGATGACCTGCACGATGCTATTCACATGGCTGCCGATAATGCAGTTCCGCACTACTACGCTGACATCTTTAGCGTAATGGCAAGTGAGGGCATTGACCTTGAGTTCGAAGACTCTGGTCTGATGCCTGACACCAAGGACGTAATCCGCATCCTGCAAGCGCGTATCTATGAGCAATTAACGATTGACCTCTGGGAAGACGCAGAAGACTTGCTCACTGAATACTTGGAGGAAGTCgaggaagtcg                          >  1:468541/1‑296 (MQ=255)`  
        `cACGCTTACGAAATGCTGAAAGAAAACATCCGTTATGATGACATCCGTGACACTGATGACCTGCACGATGCTATTCACATGGCTGCCGATAATGCAGTTCCGCACTACTACGCTGACATCTTTAGCGTAATGGCAAGTGAGGGCATTGACCTTGAGTTCGAAGACTCTGGTCTGATGCCTGACACCAAGGACGTAATCCGCATCCTGCAAGCGCGTATCTATGAGCAATTAACGATTGACCTCTGGGAAGACGCAGAAGACTTGCTCAATGAATACTTGGAGGAAGTCgaggaagtcgcac                       <  1:507714/301‑9 (MQ=255)`  
          `cGCTTACGAAATGCTGAAAGAAAACATCCGTTATGATGACATCCGTGATACTGATGACCTGCACGATGCTATTCACATGGCTGCCGATAATGCAGTTCCGCACTACTACGCTGACATCTTTAGCGTAATGGCAAGTGAGGGCATTGACCTTGAGTTCGAAGACTCTGGTCTGATGCCTGACACCAAGGACGTAATCCGCATCCTGCAAGCGCGTATCTATGAGCAATTAACGATTGACCTCTGGGAAGACGCAGAAGACTTGCTCAATGAATACTTGGAGGAAGTCgaggaagtcgcacga                     >  1:529314/1‑291 (MQ=255)`  
          `cGCTTACGAAATGCTGAAAGAAAACATCCGTTATGATGACATCCGTGACACTGATGACCTGCACGATGCTATTCACATGGCTGCCGATAATGCAGTTCCGCACTACTACGCTGACATCTTTAGCGTAATGGCACGTGAGGGCATTGACCTTGAGTTCGAAGACTCTGGTCTGATGCCTGACACCAAGGACGTAATCCGCATCCTGCAAGCGCGTATCTATGAGCAATTAACGATTGACCTCTGGGAAGACGCAGAAGACTTGCTCAATGAATACTTGGAGGAAGTCgaggaagtcgcacga                     >  1:241394/1‑291 (MQ=255)`  
          `cGCTTACGAAATGCTGAAAGAAAACATCCGTTATGATGACATCCGTGACACTGATGACCTGCACGATGCTATTCACATGGCTGCCGATAATGCAGTTCCGCACTACTACGCTGACATCTTTAGCGTAATGGCAAGTGAGGGCATTGACCTTGAGTTCGAAGACTCTGGTCTGATGCCTGACACCAAGGACGTAATCCGCATCCTGCAAGCGCGTATCTATGAGCAATTAACGATTGACCTCTGGGACGACGCAGAAGACTTGCTCAATGAATACTTGGAGGAAGTCgaggaagtcgcacga                     >  1:498459/1‑291 (MQ=255)`  
          `cGCTTACGAAATGCTGAAAGAAAACATCCGTTATGATGACATCCGTGACACTGATGACCTGCACGATGCTATTCACATGGCTGCCGATAATGCAGTTCCGCACTACTACGCTGACATCTTTAGCGTAATGGCAAGTGAGGGCATTGACCTTGAGTTCGAAGACTCTGGTCTGATGCCTGACACCAAGGACGTAATCCGCATCCTGCAAGCGCGTATCTATGAGCAATTAACGATTGACCTCTGGGAAGACGCAGAAGACTTGCTCAATGAATACTTGGAGGAAGTCgaggaagtcgcacga                     >  1:619617/1‑291 (MQ=255)`  
           `gCTTACGAAATGCTGAAAGAAAACATCCGTTATGATGACATCCGTGACACTGATGACCTGCACGATGCTATTCACATGGCTGCCGATAATGCAGTTCCGCACTACTACGCTGACATCTTTAGCGTAATGGCAAGTGAGGGCATTGACCTTGAGTTCGAAGACTCTGGTCTGATGCCTGACGCCAAGGACGTAATCCGCATCCTACAAGCGCGTATCTATGAGCAATTAACGATTGACCTCTGGGAAGACGCAGAAGACTTGCTCAATGAATACTTGGAGGAAGTCgaggaagtcgcacgac                    <  1:250562/301‑12 (MQ=255)`  
           `gCTTACGAAATGCTGAAAGAAAACATCCGTTATGATGACATCCGTGACACTGATGACCTGCACGATGCTATTCACATGGCTGCCGATAATGCAGTTCAGCACTACTACGCTGACATCTTTAGCGTAATGGCAAGTGAGGGCATTGACCTTGAGTTCGAAGACTCTGGTCTGATGCCTGACACCAAGGACGTAATCCGCATCCTGCAAGCGCGTATCTATGAGCAATTAACGATTGACCTCTGGGAAGACGCAGAAGACTTGCTCAATGAATACTTGGAGGAAGTCtaggaagtcgcacgac                    >  1:587127/1‑290 (MQ=255)`  
           `gCTTACGAAATGCTACAAGAAAACATCCGTTATGATGACATCCGTGACACTGATGACCTGCACGATGCTATTCACATGGCTGCCGATAATGCAGTTCCGCACTACTACGCTGACATCTTTAGCGTAATGGCAAGTGAGGGCATTGACCTTGAGTTAGAAGACTCTGGTCTGATGCCTGACACCAAGGACGTAATCCGCATCCTGCAAGCGCGTATCTATGAGCAATTAACGATTGACCTCTGGGAAGACGCAGAAGACTTGCTCAATGAATACTTGGAGGAAGTCgaggaagtcgcacgac                    <  1:478045/301‑12 (MQ=255)`  
           `gCTGACGTAATGCTGAAAGAAAACATCCGTTATGATGACATCCGTGACACTGATGACCTGCACGATGCGATTCACATGGCTGCCGATAATGCAGTTCCGAACTACGACGCTGACATCTTTAGCGTAATGGCAAGTGAGGGCATTGACCTTGAGTTCGAAGACTCTGGTCTGATGCCTGACACCAAGGACGTAATCCGCATCCTGCAAGCGCGTATCTATGAGCAATTAACGATTGACCTCTGGGAAGACGCAGAAGACTTGCTCAATGAATACTTGGAGGAAGTCgaggaagtcgcacgac                    <  1:73462/301‑12 (MQ=255)`  
            `cTTACGAAATGCTGAAAGAAAACATCCGTTATGATGACATCCGTGACACTGATGACCTGCACGATGCTATTCACATGGCTGCCGATAATGCAGTTCCGCACTACTACGCTGACATCTTTAGCGTAATGGCAAGTGAGGGCATTGACCTTGAGTTCGAAGACTCTGGTCTGATGCCTGACACCAAGGACGTAATCCGCATCCTGCAAGCGCGTATCTATGAGCAATTAACGATTGACCTCTGGGAAGACGAAGAAGACTTGCTCAATGAATACTTGGAGGAAGTCgaggaagtcgcacgaca                   >  1:84762/1‑289 (MQ=255)`  
             `ttACGAAATGCTGAAAGAAAACATCCGTTATGATGACATCCGTGACACTGATGACCTGCACGATGCTATTCACATGGCTGCCGATAATGCAGTTCCGCACTACTACGCTGACATCTTTAGCGTAATGGCAAGTGAGGGCATTGACCTTGAGTTCGAAGACTCTGGTCTGATGCCTGACACCAAGGACGTAATCCGCATCCTGCAAGCGCGTATCTATGAGCAATTAACGATTGACCTCTGGGAAGACGCAGAAGACTTGCTCAATGAATACTTGGAGGAAGTCgaggaagtcgcacgacag                  <  1:82066/301‑14 (MQ=255)`  
                  `aaaTGCTGAAAGAAAACATCCGTTATGATGACATCCGTGACACTGATGACCTGCACGATGCTATTCACATGGCTGCCGATAATGCAGTTCCGCACTACTACGCTGACATCTTTAGCGTAATGGCAAGTGAGGGCATTGACCTTGAGTTCGAAGACTCTGGTCTGATGCCTGACACCAAGGACGTAATCCGCATCCTGCAAGCGCGTATCTATGAGCAATTAACGATTGACCTCTGGGAAGACGCAGAAGACTTGCTCAATGAATACTTGGAGGAAGTCgaggaagtcgcacgacagaaaga             <  1:459536/301‑19 (MQ=255)`  
                  `aaaTGCTGAAAGAAAACAGCCGTTATGATGACAGCCGTGACAATGAGGACCTGAACGATGCTATTCACATGGCTGCCGATAATGCAGTTCCGCACTACTACGCTGACATCTTTAGCGTAATGGCAAGTGAGGGCATTGACCTTGAGTTCGAAGACGCTGGTCTGATGCCTGACACCAAGGACGTAATCCGCATCCTGCAAGCGCGTATCTATGAGCAATTAACGATTGACCTCTGGGAAGACGCAGAAGACTTGCTCAATGAATACTTGGAGGAAGTCgaggaagtcgcacgacagaaaga             <  1:217581/301‑19 (MQ=255)`  
                    `aTGCTGAAAGAAAACATCCGTTATGATGACATCCGTGACACTGATGACCTGCACGATGCTATTCACATGGCTGCCGATAATGCAGTTCCGCACTACTACGCTGACATCTTTAGCGTAATGGCAAGTGAGGGCATTGACCTTGAGTTCGAAGACTCTGGTCTGATGCCTGACACCAAGGACGTAATCCGCATCCTGCAAGCGCGTATCTATGAGCAATTAACGATTGACCTCTGGGAAGACGCAGAAGACTTGCTCAATGAATACTTGGAGGAAGTCgaggaagtcgcacgacagaaagaaa           >  1:55883/1‑281 (MQ=255)`  
                     `tGCTGAAAGAAAACATCCGTTATGATGACATCCGTGACACTGATGACCTGCACGATGCTATTCACATGGCTGCCGATAATGCAGTTCCGCACTACTACGCTGACATCTTTAGCGTAATGGCAAGTGATGGCATTGACCTTGAGTTCGAAGACTCTGGTCTGATGCCTTACACCAAGGACGTAATCCGCATCCTGCAAGCGCGTATCTATGAGCAATTAACGATTGACCTCTGGGAAGACGCAGAAGACTTGCTCAATGAATACTTGGAGGAAGTCgaggaagtcgcacgacagaaagaaat          <  1:173265/301‑22 (MQ=255)`  
                     `tGCTGAAAGAAAACATCCGTTATGATGACATCCGTGACACTGATGACCTGCACGATGCTATTCACATGGCTGCCGATAATGCAGTTCCGCACTACTACGCTGACATCTTTAGCGTAATGGCAAGTGAGGGCATTGACCTTGAGTTCGAAGACTCTGGTCTGATGCCTGACACCAAGGACGTAATCCGCATCCTGCAAGCGCGTATCTATGAGCAATTAACGATTGACCTCTGGGAAGACGCAGAAGACTTGCTCAATGAATACTTGGAGGAAGTTgaggaagtcgcacgacagaaagaaat          <  1:540013/301‑22 (MQ=255)`  
                     `tGCTGAAAGAAAACATCCGTTATGATGACATCCGTGACACTGATGACCTGCACGATGCTATTCACATGGCTGCCGATAATGCAGTTCCGCACTACTACGCTGACATCTTTAGCGTAATGGCAAGTGAGGGCATTGACCTTGAGTTCGAAGACTCTGGTCTGATGCCTGACACCAAGGACGTAATCCGCATCCTGCAAGCGCGTATCTATGAGCAATTAACGATTGACCTCTGGGAAGACGCAGAAGACTTGCTCAATGAATACTTGGAGGAAGTCgaggaagtcgcacgacagaaagaaat          <  1:354053/301‑22 (MQ=255)`  
                     `tGCTGAAAGAAAACATCCGTTATGATGACATCCGTGACACTGATGACCTGCACGATGCTATTCACATGGCTGCCGATAATGCAGTTCCGCACTACTACGCTGACATCTTTAGCGTAATGGCAAGTGAGGGCATTGACCTTGAGTTCGAAGACTCTGGTCTGATGCCTGACACCAAGGACGTAATCCGCATCCTGCAAGCGCGTATCTATGAGCAATTAACGATTGACCTCTGGGAAGACGCAGAAGACTTGCTCAATGAATACTTGGAGGAAGTCgaggaagtcgcacgacagaaagaaat          <  1:269590/301‑22 (MQ=255)`  
                     `tGCTGAAAGAAAACATCCGTGATGATGACATCCGTGACACTGATGACCTGCACGATGCTATTCACATGGCTGCCGATAATGCAGTTCCGCACTACTACGCTGACATCTTTAGCGTAATGGCAAGTGAGGGCATTGACCTTGAGTTCGAAGACTCTGGTCTGATGCCTGACACCAAGGACGTAATCCGCATCCTGCAAGCGCGTATCTATGAGCAATTAACGATTGACCTCTGGGAAGACGCAGAAGACTTGCTCAATGAATACTTGGAGGAAGTCgaggaagtcgcacgacagaaagaaat          <  1:603716/301‑22 (MQ=255)`  
                     `aagagacagaaaACAACCGTTAGGATAACATCCGTGACACTGATGACCTGCACGATGCTATTCACAGGGATGCCGATAATGCAGTTCCGCACTACTACGCTGACATCTTTAGCGTAATGGAAAGTGAGGGCATTGACCTTGAGGTCGAAGACTCTGGTCTGATGCCTGACACCAAGGACGTAATCCGCATCCTGCAAGCGCGTATCTATGAGCAATTAACGATTGACCTCTGGGAAGACGCAGAAGACTTGCTCAATGAATACTTGGAGGAAGTCgaggaagtcgcacgacagaaagaaat          <  1:223838/294‑22 (MQ=255)`  
                       `cTGAAAGAAAACCTCCGTTATGATGACATCCGTGACACTGATGACCTGCACGATGCTATTCACATGGCTGCCGATAATGCAGTTCCGCACTACTACGCTGACATCTTTAGCGTAATGGCAAGTGAGGGCATTGACCTTGAGTTCGAAGACTCTGGTCTGATGCCTGACACCAAGGACGTAATCCGCATCCTGCAAGCGCGTATCTATGAGCAATTAACGATTGACCTCTGGGAAGACGCAGAAGACTTGCTCAATGAATACTTGGAGGAAGTCgaggaagtcgcacgacagaaagaaattg        <  1:495910/301‑24 (MQ=255)`  
                         `gaaagaaaACATCCGTTATGATGACATCCGTTACACTGATGACCTGCACGATGCTATTCACATGGATGCCGATAATGCAGTTCCGCACTACTACGCTGACATCTTTAGCGTAATGGCAAGTGAGGGCATTGACCTTGAGTTCGAAGACTCTGGTCTGATGCCTGACACCAAGGACGTAATCCGCATCCTGCAAGCGCGTATCTATGAGCAATTAACGATTGACCTCTGGGAAGACGCAGAAGACTTGCTCAATGAATACTTGGAGGAAGTCgaggaagtcgcacgacagaaagaaattgac      <  1:342419/301‑26 (MQ=255)`  
                         `gaaagaaaACATCCGTTATGATGACATCCGTGACACTGATGACCTGCACGATGCTATTCACATGGCTGCCGATAATGCAGTTCCGCACTACTACGCTGACATCTTTAGCGTAATGGCAAGTGAGGGCATTGACCTTGAGTTCGAAGACTCTGGTCTGATGCCTGACACCAAGGACGTAATCCGCATCCTGCAAGCGCGTATCTATGAGCAATTAACGATTGACCTCTGGGAAGACGCAGAAGACTTGCTCAATGAATACTTGGAGGAAGTCgaggaagtcgcacgacagaaagaaattgac      <  1:138058/301‑26 (MQ=255)`  
                         `gaaagaaaACATCCGTTATGATGACATCCGTGACACTGATGACCTGCACGATGCTATTCACATGGCTGCCGATAATGCAGTTCCGCACTACTACGCTGACATCTTTAGCGTAATGGCAAGTGAGGGCATTGACCTTGAGTTCGAAGACTCTGGTCTGATGCCTGACACCAAGGACGTAATCCGCATCCTGCAAGCGCGTATCTATGAGCAATTAACGATTGACCTCTGGGAAGACGCAGAAGACTTGCTCAATGAATACTTGGAGGAAGTCgaggaagtcgcacgacagaaagaaattgac      >  1:34252/1‑276 (MQ=255)`  
                         `gaaagaaaACATCCGTTATGATGACATCCGGGACACTGATGACCGGCACGATGCTAGTCAGATGGCGGCCGATAATGCAGTGCCGCACTACGACGCTGACATCTTTAGCGTAATGGCAAGTGAGGGCATGGACCTTGAGTTCGAAGACTCTGGTCTGATGTCTGACACCAAGGACGTAATCCGCATCCTGCAAGCGCGTATCTATGAGCAATTAACGATTGACCTCTGGGAAGACGCAGAAGACTTGCTCAATGAATACTTGGAGGAAGTCgaggaagtcgcacgacagaaagaaattgac      <  1:427819/301‑26 (MQ=255)`  
                         `acaggaaaACATCCGTTATGATGACATCCGTGACAATGATGACCTGCACGATGCTATTCACATGGCTGCCGATAATGCAGTTCCGCACTACTACGCTGACATCTTTAGCGTAATGGCAAGTGAGGGCATTGACCTTGAGTTCGAAGACTCTGGTCTGATGCCTGACACCAAGGACGTAATCCGCATCCTGCAAGCGCGTATCTATGAGCAATTAACGATTGACCTCTGGGAAGACGCAGAAGACTTGCTCAATGAATACTTGGAGGAAGTCgaggaaatcgcacgacagaaagaaattgac      <  1:354899/297‑26 (MQ=255)`  
                         `aaaagaaaACATCCGTTATGATGACATCCGTGACACTGATGACCTGCACGATGCTATTCACATGGCTGCCGATAATGCAGTTCCGCACTACTACGCTGACATCTTTAGCGTAATGGCAAGTGAGGGCATTGACCTTGAGTTCGAAGACTCTGGTCTGATGCCTGACACCAAGGACGTAATCCGCATCCTGCAAGCGCGTATCTATGAGCAATTAACGATTGACCTCTGGGAAGACGCAGAAGACTTGCTCAATGAATACTTGGAGGAAGTCgaggaagtcgcacgacagaaagaaattgac      <  1:367518/300‑26 (MQ=255)`  
                         `aaaagaaaACATCCGTTATGATGACATCCGTAACACTGATGACCTGCACGATGCTATTCACATGGCTGCCGATAATGCAGTTCCGCACTACTACGCTGACATCTTTAGCGTAATGGCAAGTGAGGGCATTGACCTTGAGTTCGAAGACTCTGGTCTGATGCCTGACACCAAGGACGTAATCCGCATCCTGCAAGCGCGTATCTATGAGCAATTAACGATTGACCTCTGGGAAGACGCAGAAGACTTGCTCAATGAATACTTGGAGGAAGTCgaggaagtcgcacgacagaaagaaattgac      <  1:543909/300‑26 (MQ=255)`  
                         `aaaagaaaACATCCGTTATGATGACATCCGGGACACTGATGACCTGCACGAGGCTATTCACATGGCTGCCGATAATGCAGTTCCGCACTACTACGCTGACATCTTTAGCGTAATGGCAAGTGAGGGCATTGACCTTGAGTTCGAAGACTCTGGTCTGATGCCTGACACCAAGGACGTAATCCGCATCCTGCAAGCGCGTATCTATGAGCAATTAACGATTGACCTCTGGGAAGACGCAGAAGACTTGCTCAATGAATACTTGGAGGAAGTCgaggaagtcgcacgacagaaagaaattgac      <  1:654200/300‑26 (MQ=255)`  
                             `gaaaACATCCGTTATGATGACATCCGTGACACTGATGACCTGCACGATGCTATTCACATGGCTGCCGATAATGCAGTTCCGCACTACTACGTTGACATCTTTAGCGTAATGGCAAGTGAGGGCATTGACCTTGAGTTCGAAGACTCTGGTCTGATGCCTGACACCAAGGACGTAATCCGCATCCTGCAAGCTCGTATCTATGAGCAATTAACGATTGACCTCTGGGAAGACGCAGAAGACTTGCTCAATGAATACTTGGAGGAAGTCgaggaagtcgcacgacagaaagaaattgaccgcg  >  1:389090/1‑272 (MQ=255)`  
                             `gaaaACATCCGTTATGATGACATCCGTGACACTGATGACCTGCACGATGCTATTCACATGGCTGCCGATAATGCAGTTCCGCACTACTACGCTGACATCTTTAGCGTAATGGCAAGTGAGGGCATTGACCTTGAGTTCGAAGACTCTGGTCTGATGCCTGACACCAAGGACGTAATCCGCATCCTGCAAGCGCGTATCTATGAGCAATTAACGATTGACCTCTGGGAAGACGCAGAAGACTTGCTCAATGAATACTTGGAGGAAGTCgaggaagtcgcacgacctgtctcttatacacatc  >  1:110960/1‑272 (MQ=255)`  
                             `gaaaACATCCGTTATGATGACATCCGTGACACTGATGACCTGCACGATGCTATTCACATGGCTGCCGATAATGCAGTTCCGCACTACTACGCTGACATCTTTAGCGTAATGGCAAGTGAGGGCATTGACCTTGAGTTCGAAGACTCTGGTCTGATGCCTGACACCAAGGACGTAATCCGCATCCTGCAAGCGCGTATCTATGAGCAATTAACGATTGACCTCTGGGAAGACGCAGAAGACTTGCTCAATGAATACTTGGAGGAAGTCgaggaagtcgcacgacagaaagaaattgaccgcg  >  1:202605/1‑272 (MQ=255)`  
                             `aaaaaCATCCGTTATGATGACATCCATGAAACTGATGACCTGCACGATGCTATTCACATGGCTGCCGATAATGCAGTTCCGCACTACTACGCTGACATCTTTAGCGTAATGGCAAGTGAGGGCATTGACCTTGAGTTCGAAGACTATGGTCTGATGCCTGACACCAAGGACGTAATCCGCATCCTGCAAGCGCGTATCTATGAGCAATTAACGATTGACCTCTGGGAAGACGCAGAAGACTTGCTCAATGAATACTTGGAGGAAGTCgaggaagtcgcacgacagaaagaaattgaccgcg  <  1:385773/300‑30 (MQ=255)`  

                                                                                                                                                                                                                                                                                                            `|`                               
`TTTTCGACCACGCTTACGAAATGCTGAAAGAAAACATCCGTTATGATGACATCCGTGACACTGATGACCTGCACGATGCTATTCACATGGCTGCCGATAATGCAGTTCCGCACTACTACGCTGACATCTTTAGCGTAATGGCAAGTGAGGGCATTGACCTTGAGTTCGAAGACTCTGGTCTGATGCCTGACACCAAGGACGTAATCCGCATCCTGCAAGCGCGTATCTATGAGCAATTAACGATTGACCTCTGGGAAGACGCAGAAGACTTGCTCAATGAATACTTGGAGGAAGTCGAGGAGTACGAGGAGGATGAAGAGTAATGTCTAC  >  NC_001604.1/956‑1285`

  

Alignment Legend

Aligned base mismatch/match (shaded by quality score): `ATCG/ATCG` `< 3 ≤` `ATCG/ATCG` `< 11 ≤` `ATCG/ATCG` `< 23 ≤` `ATCG/ATCG` `< 35 ≤` `ATCG/ATCG` `< 38 ≤` `ATCG/ATCG`

Unaligned base: `atcg`    Masked matching base: `atcg`    Alignment gap: `‑`    Deleted base: `‑`

Reads not counted as support for junction

read\_name Not counted due to insufficient overlap past the breakpoint.

read\_name Not counted due to not crossing MOB target site duplication.
